# The role of ultrasound quantitative parameters in the assessment of acute radiodermatitis after breast-conserving surgery

**DOI:** 10.1093/jrr/rrad029

**Published:** 2023-05-08

**Authors:** Wenqin Chen, Wenjuan Lu, Ya Yuan, Lu Li, Hongyan Deng, Xinhua Ye

**Affiliations:** Department of Ultrasound, The First Affiliated Hospital of Nanjing Medical University, 300 Guangzhou Road, Nanjing, Jiangsu 210029, China; Department of Ultrasound, The First Affiliated Hospital of Nanjing Medical University, 300 Guangzhou Road, Nanjing, Jiangsu 210029, China; Department of Ultrasound, The First Affiliated Hospital of Nanjing Medical University, 300 Guangzhou Road, Nanjing, Jiangsu 210029, China; Department of Ultrasound, The First Affiliated Hospital of Nanjing Medical University, 300 Guangzhou Road, Nanjing, Jiangsu 210029, China; Department of Ultrasound, The First Affiliated Hospital of Nanjing Medical University, 300 Guangzhou Road, Nanjing, Jiangsu 210029, China; Department of Ultrasound, The First Affiliated Hospital of Nanjing Medical University, 300 Guangzhou Road, Nanjing, Jiangsu 210029, China

**Keywords:** breast-conserving surgery, radiotherapy, radiodermatitis, ultrasound, ultrasound elastography

## Abstract

This study aimed to assess the severity of acute radiodermatitis (ARD) by ultrasound quantitative parameters and to try to identify the influencing factors of skin toxicity. A total of 55 patients who underwent radiotherapy after unilateral breast-conserving surgery (BCS) were included in the study. The irradiated side of the breast was used as the research object and the quantitative ultrasound parameters (skin thickness, shear wave elasticity) were evaluated before radiotherapy, every week during radiotherapy. Two weeks after radiotherapy, the patients were divided into two groups, according to the World Health Organization scoring standard: mild (0–2 grade) and severe (3–4 grade). The differences in the parameters between the groups and the changes during radiotherapy were compared, and the relationship between these parameters and the severity of ARD was analyzed. In addition, some clinical factors that may affect ARD were also included in our study. Ninety-eight percent of patients developed different degrees of ARD, and Group 2 accounted for ~31%. At the end of 5 weeks of radiotherapy, the difference in thickness between the two groups was statistically significant (*P* < 0.05). There was no significant change in the elastic modulus of breast skin between the two groups (*P* > 0.05). Body mass index >25 kg/m^2^, breast thickness ≥18 mm, skin basic elastic modulus <23 kPa and skin thickness increment >0.3 mm were considered to be associated with severe skin reactions (*P* < 0.05). Ultrasound can be a useful tool for the non-invasive and objective assessment of skin changes during radiotherapy, documenting quantitative changes in the skin of breast cancer patients following BCS undergoing radiotherapy.

## INTRODUCTION

Radiotherapy has been widely used as a surgical procedure after breast-conserving surgery (BCS) for early breast cancer, and its 10-year overall survival has been shown to be equivalent to total mastectomy [1]. In this case, the most common side effect is acute radiation skin damage [2].

Acute radiodermatitis (ARD), also known as skin toxicity, occurs between hours and weeks after radiotherapy, peaking at 1–2 weeks after radiotherapy is completed, which would cause skin erythema, edema, dry or moist scaling, burning sensation and bleeding [3–5]. The pathogenesis of radiodermatitis is due to cellular damage and an inflammatory response affecting the layers of the skin [6, 7]. When severe skin lesions such as wet desquamation occur, it may lead to impaired skin barrier function, bacterial colonization, superinfection and superantigen production [8]. In some patients, radiotherapy may be interrupted during the treatment, potentially reducing its efficacy and affecting the patient’s quality of life [3, 7, 9].

ARD is commonly assessed clinically using criteria developed by the Radiation Therapy Oncology Group (RTOG) or the National Cancer Institute's Common Terminology Criteria for Adverse Events (CTCAE), in agreement with the World Health Organization (WHO) and the Society for Nursing Oncology scales. Visual assessment of skin condition is performed subjectively by the examining physician, and it is well known that estimates of visible changes can be significantly biased by different examiners. Studies have pointed to low overall agreement between raters when using RTOG and CTCAE scales [10]. Therefore, we tried to find a quantitative assessment method to assess ARD.

Ultrasound can reliably measure skin thickness with good inter- and intra-observer reproducibility [11]. High-frequency ultrasound is widely used in dermatology to measure skin thickness to assess scleroderma or skin fibrosis. Several studies suggest that ultrasound has the potential to quantify the severity of ARD by measuring the increase in skin thickness in the irradiated area [12–14]. Ultrasound elastography is a non-invasive, cost-effective, safe and widely used new technology that can provide more elasticity information [15]. Some scholars have used the average elasticity ratio of the subcutaneous tissue after radiotherapy to assess the edema of the subcutaneous tissue [16]. Shear wave elastography (SWE) uses a sequence of acoustic radiation force pulses to generate shear waves that are perpendicular to the propagation of the ultrasonic beam causing instantaneous displacement. Automatic registration of shear wave images with standard B-mode images to provide anatomical specific quantitative color elastograms plays a good role in determining the severity of the disease and the treatment and follow-up of various soft tissues [17].

Given these reported findings, this study was designed to evaluate the usefulness of ultrasound as quantitative measures of radiation response in preserved breast skin, and to try to find influencing factors for ARD.

## PATIENTS AND METHODS

The study population consisted of women who were scheduled to undergo postoperative breast-conserving radiotherapy in the First Affiliated Hospital of Nanjing Medical University. The inclusion criteria were: (i) patients with single breast cancer on one side of the breast (To reduce errors, we selected patients with tumors in the upper quadrant of the breast.) and (ii) a radiotherapy dose of 50 Gy/25 fractions (F) for the whole breast on the affected side and 10 Gy/5F for the tumor bed area, by using 6 MV photons.

**Fig. 1:**
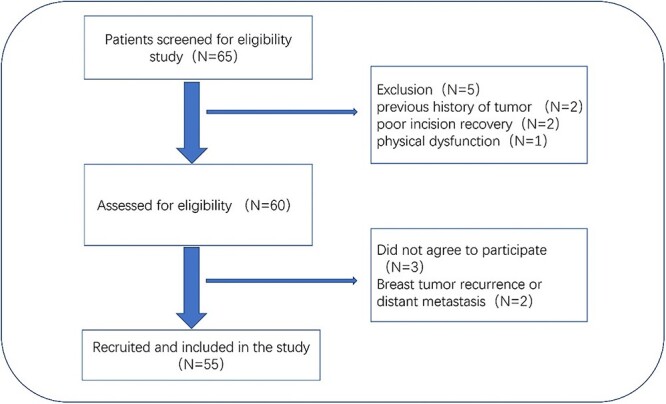
Patient recruitment pathway flowchart. Primary unilateral breast cancer; BCS; radiotherapy planned.

Exclusion criteria: bilateral breast cancer; patients requiring irradiation of regional lymph nodes; multi-center disease; evidence of distant metastasis; severe cardiopulmonary diseases, mental illness and physical dysfunction; previous history of tumor and/or chest irradiation; poor incision recovery; skin tumors and other genetic disorders involving the skin in the treatment area (Fig. 1).

The study involved a non-invasive and painless ultrasound examination in addition to usual care and was approved by the local ethics committee; the study followed the rules of the Declaration of Helsinki. Patients gave their informed consent to be in the study.

### Skin toxicity assessment

The skin condition of the patient was evaluated by two experienced radiologists. Evaluation of ARD occurred every week during radiotherapy, and 2 weeks after treatment. We assessed radiodermatitis according to the WHO scale. Grade 0: no change; Grade 1: erythema; Grade 2: dry scaling, pruritus; Grade 3: wet scaling, ulceration; Grade 4: ulcerative necrosis, skin necrosis requiring surgical intervention. Because the skin ulcer causes severe pain and other discomfort, patients were divided into two groups based on the most severe skin reaction during the observation period, with Grades 0–2 reactions as Group 1 and Grades 3–4 reactions as Group 2. It is worth noting that in some studies, wet scaling at skin folds is classified as a second-order reaction, and it is believed that such scaling is caused by excessive friction at folds due to large breast volume. In this study, the breast volume was considered as an influencing factor and the obvious wet scaling at the skin folds was classified as a Grade 3 reaction.

### Ultrasound imaging

Ultrasonography was performed before radiotherapy and every week during radiotherapy, approximately at the same time as skin toxicity assessments. Two experienced sonographers performed the ultrasound examination, and they had good interclass consistency and good intraclass consistency (Table 1). The machine (L15–4 linear-array transducer; Supersonics Aixplorer; Supersonic Imagine; France) and settings were used for all examinations, including gain, depth and frequency. Exams are performed at room temperature of 20–25°C with the patient supine, hands raised above the head and the ultrasound scans are performed by an experienced sonographer. Standard echo gel is used as a couplant between the skin surface and the probe. To ensure good coupling of the probe/skin interface, in addition to the gel, a sound-guiding pad with a size of 130 × 120 × 10 mm was used. During all examinations, special care was taken to avoid any pressure on the skin surface, and the probe is gently applied to the surface of the skin.

**Table 1:** Consistency of the observers with regard to ultrasound parameters

	1	2	3	ICC (95% CI)
Skin thickness (mm)	2.13 ± 0.35	2.10 ± 0.28	2.12 ± 0.26	0.919 (0.878–0.949)
Elastic modulus (kpa)	20.04 ± 6.87	20.63 ± 4.95	19.47 ± 5.26	0.858 (0.789–0.909)
	Observer A	Observer B		CCC (95% CI)
Skin thickness (mm)	2.13 ± 0.35	2.07 ± 0.23		0.834 (0.732–0.900)
Elastic modulus (kpa)	20.04 ± 6.87	19.73 ± 5.05		0.874 (0.794–0.925)

The ICC and the CCC were used to examine the inter- and intra-observer agreement of repetition elasticity and skin thickness.

**Fig. 2:**
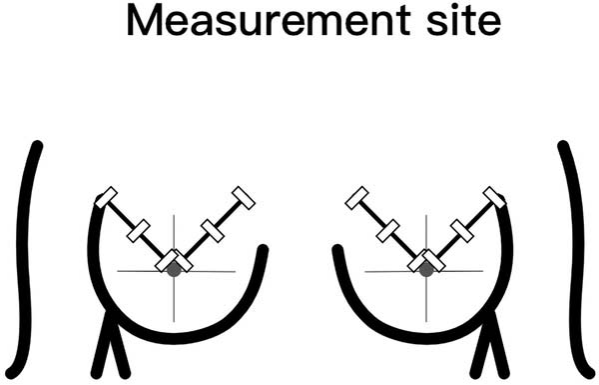
Measurement points for ultrasonic examination. The three measurement points in the tumor quadrant and the mirror quadrant of the opposite breast are measured, respectively, and the average value of the three measurement points is.

**Fig. 3:**
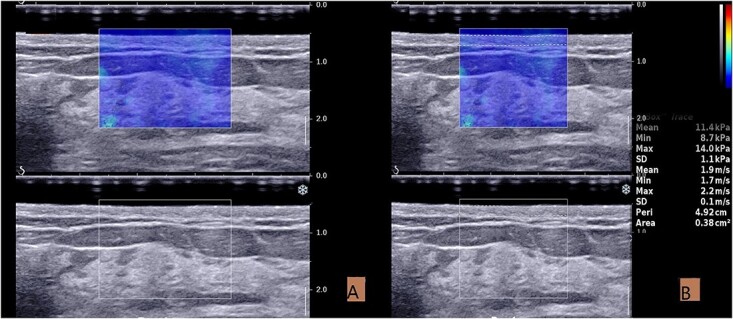
Acquisition and measurement of ultrasound images. (**A**) The SWE image taken in the resting state. (**B**) Measurement of elastic modulus in SWE mode. The skin boundary is traced manually to measure the elastic value of the entire skin layer.

On the surgical quadrant junction of the radiating breast, the outer edge of the areola is the first measurement point, and the other two measurement points are ~3 cm apart (Fig. 2). The measurement site should be atleast 1 cm away from the surgical incision. After acquiring the B-mode ultrasound image, switch directly to the SWE mode. Once the image is stable, use the Q-BOX manual tracing function to outline the entire skin layer to obtain elasticity parameters (Fig. 3). All quantitative data consist of the mean of three measurement points. In addition, the skin elasticity of the mirror quadrant breast was used as the patient’s breast skin elasticity baseline, and breast thickness (includes skin layer, subcutaneous fat layer and gland layer) adjacent to the nipple was used as the patient’s breast thickness.

### Data analysis

We used spss24.0 for statistical analysis of the data. Descriptive statistics such as frequency, mean, standard deviation and percentage were used to characterize patients. A normal distribution is assumed using the Shapiro–Wilk test. For univariate comparisons of baseline and subsequent measures, matched paired *t*-tests were used. Independent *t*-tests were performed to examine differences in breast measurements between groups. The area under receiver operating characteristic (ROC) curve analysis was used to select cutoff values. Mann–Whitney U test and Friedman test are used for those that do not fit a normal distribution. The intraclass correlation coefficient (ICC) and the interclass correlation coefficient (CCC) were applied to examine the inter- and intra-observer agreement of repetition elasticity and skin thickness. *P* < 0.05 indicated that the difference was statistically significant.

## RESULTS

### Clinical features of patients

**Fig. 4:**
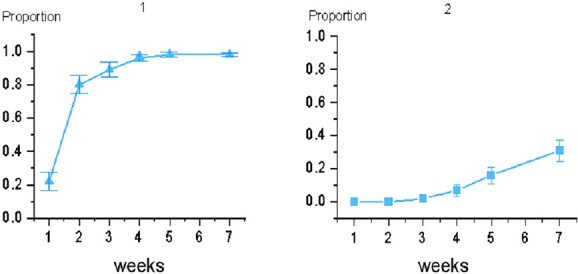
Incidence of acute toxic reactions in breast-conserving patients. Picture 1 shows the proportion of Grade 1 and higher; picture 2 shows the proportion of Grade 3.

Among 55 patients undergoing radiotherapy, 98% developed ARD of varying degrees, with a mean onset time of 2.07 weeks (standard deviation = 0.89). Among them, 67% patients (Group 1) presented with mild skin reactions; 31% patients (Group 2) presented with severe skin reactions characterized by wet desquamation. Wet desquamation begins at Week 3 and increases in frequency over the next few weeks, peaking at 2 weeks after radiotherapy. No patients developed Grade 4 skin reaction in this study (Table 2, Fig. 4).

**Table 2:** Incidence of acute toxicity in breast-conserving patients

Grade	Week					
	w1	w2	w3	w4	w5	w7
0	43	11	6	2	1	1
1	12	44	36	44	33	21
2	0	0	2	5	12	16
3	0	0	1	4	9	17
Proportion of Grade 1 or higher (95% CI)	0.22 (0.11–0.33)	0.80 (0.69–0.91)	0.89 (0.80–0.97)	0.96 (0.91–1)	0.98 (0.94–1)	0.98 (0.95–1)
Proportion of Grade 3 (95% CI)	0	0	0.02 (−0.02 to 0.06)	0.07 (0–0.14)	0.16 (0.06–0.27)	0.31 (0.18–0.44)

Proportions of Grade 1 or higher show prevalence of varying degrees of ARD.

Proportions of Grade 3 (and higher, but no Grade 4 in this study) show more severe ARD occurring later in radiotherapy.

The clinical characteristics of the two groups of subjects are shown in Table 3, among which body mass index (BMI), breast thickness and breast skin elasticity were statistically significant (*P* < 0.05). There was no statistical difference between the two groups in age, time interval from surgery to radiotherapy, tumor location, reproductive history, breastfeeding history, etc. (*P* > 0.05) (Table 3).

**Table 3:** Clinical characteristics of the two groups of patients before radiotherapy

	Group 1	Group 2	*P*
Age (year)	43.58 ± 8.41	43.53 ± 7.63	0.98
BMI ≤25 kg/m^2^ >25 kg/m^2^	34 (75.6) 4 (40.0)	11 (24.4) 6 (60.0)	0.02
Breast thickness (mm)	17.00 ± 4.37	20.35 ± 4.38	0.01
Breast skin elasticity baseline (kpa)	18.73 ± 5.21	15.10 ± 3.40	0.02
Post-operation (week)	14.91 ± 7.79	14.28 ± 7.98	0.78
Tumor location Medial Lateral	13 (65.0) 25 (71.4)	7 (35.0) 10 (28.6)	0.87
Chemotherapy Yes No	22 (68.8) 16 (69.6)	10 (31.2) 7 (30.4)	0.94
Reproduction Yes No	34 (69.4) 4 (66.7)	15 (30.6) 2 (33.3)	0.89
Breastfeeding Yes No	32 (69.6) 6 (66.7)	14 (30.4) 3 (33.3)	0.86

### Changes in ultrasound quantitative parameters

**Fig. 5:**
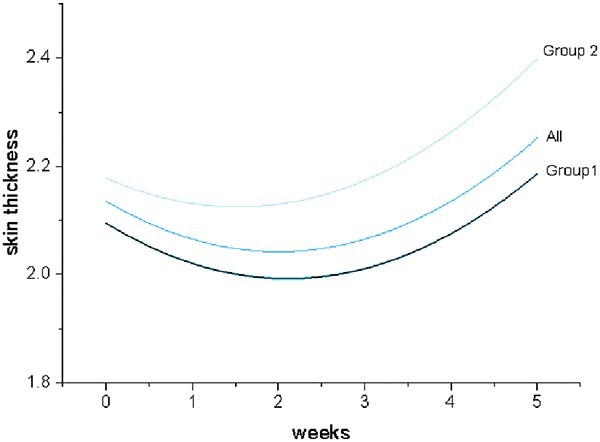
Fitting curve of weekly skin thickness changes during radiotherapy.

Before radiotherapy, there was no significant difference in skin thickness and skin elastic modulus between the two groups of patients (*P* > 0.05). During radiotherapy, skin thickness was thinner at Week 2 (*P* < 0.05) and thicker at Week 5 (*P* < 0.05) (Fig. 5). The difference is that the skin thickness in Group 2 (2.46 ± 0.51 mm) was significantly greater than that in Group 1 (2.19 ± 0.42 mm) after 5 weeks of radiotherapy (*P* < 0.05). At the same time, the skin thickness increment in Group 2 was significantly higher than that in Group 1 (*P* < 0.05), but there was no significant difference in the skin elastic modulus between the two groups during radiotherapy (Table 4).

**Table 4:** Changes of weekly ultrasound quantitative parameters during radiotherapy

Week	Skin thickness (mm)			Elastic modulus (kpa)		
	Group 1	Group 2	*P*	Group 1	Group 2	*P*
W0	2.14 ± 0.38	2.08 ± 0.27	0.61	20.25 ± 7.80	19.00 ± 4.99	0.54
W1	2.05 ± 0.36	2.23 ± 0.47	0.11	20.40 ± 8.06	18.06 ± 6.26	0.34
W2	1.96 ± 0.32	2.13 ± 0.43	0.11	20.95 ± 9.04	18.06 ± 4.39	0.21
W3	2.04 ± 0.36	2.16 ± 0.50	0.32	19.73 ± 7.78	17.02 ± 5.62	0.20
W4	2.06 ± 0.40	2.21 ± 0.38	0.18	21.00 ± 7.67	18.54 ± 3.77	0.21
W5	2.19 ± 0.42	2.46 ± 0.51	0.04	22.15 ± 9.05	19.86 ± 7.24	0.36
Increment	0.05 ± 0.35	0.38 ± 0.52	0.02	1.90 ± 8.49	0.87 ± 8.49	0.679

Increment: Increment of w5 over w0.

### Correlation analysis

When using continuous variables, skin thickness increment was weakly positively correlated with ARD severity (*r* = 0.281, *P* = 0.037); when using dichotomous variables, skin thickness increment >0.3 mm was moderately positively correlated with ARD severity (*r* = 0.351, *P* = 0.009). In addition, the ROC curve indicated that BMI >25 kg/m^2^ [odds ratio (OR) = 5.76,95% confidence interval (CI) 1.42–23.40, *P* = 0.009], breast thickness ≥18 mm (OR = 4.63,95%CI 1.10–19.49, *P* = 0.036) and breast skin elastic modulus <23 kPa (OR = 1.58,95% CI 1.27–1.97, *P* = 0.029) are suitable cut-off values for more severe ARD (Table 5).

**Table 5:** Univariate regression analysis

Factors	OR	95% CI	*P*
BMI > 25 kg/m^2^	5.76	1.42–23.40	0.009
Breast thickness ≥ 18 mm	4.63	1.10–19.49	0.036
Skin elasticity baseline < 23 kpa	1.58	1.27–1.97	0.029
Thickness increment > 0.3 mm	4.98	1.41–17.50	0.009

## DISCUSSION

Ionizing radiation is an important tool in the treatment of early-stage breast cancer, but it often has pathological effects on normal tissue. Since tissue radiosensitivity relies on cell proliferation, tissues with greater regenerative capacity, such as skin, will be more susceptible to radiation [7]. Scholars have pointed out that radiodermatitis had the greatest negative impact on the quality of life of women with breast cancer [18, 19], especially regarding all aspects of skin-related quality of life, which significantly worsen between before and 5 weeks of radiotherapy [3].

In our study, the proportion of patients with ARD reached 98% after 5 weeks of radiotherapy, which is consistent with other international literature [3]. Various topical or systemic drugs are currently clinically tested to prevent or treat dermatitis, but quantitatively reliable and confirmatory data are scarce and not yet unified.

Quantitative methods that have been used to monitor skin changes after radiotherapy include measuring skin erythema by optical means, assessing skin water content by measuring its dielectric constant and examining skin thickness and microcirculatory blood flow by ultrasound imaging [14, 20, 21]. Among the many methods, ultrasound has been shown to be an accurate and validated method for many skin evaluation studies after radiotherapy [22–24].

In this study, quantitative ultrasound was demonstrated as an objective means of assessing acute cutaneous toxicity following radiotherapy in breast-conserving breast cancer patients. We found that the skin thickness showed a trend of first thinning and then thickening during radiotherapy. Skin thickness showed a similar trend in previous studies [22, 25].

When the breast received 20 Gy of radiation, patients developed ARD with erythema as the main symptom, which develops in response to damage to the basal layer of the skin and due to inflammatory processes [26, 27]. Keratinocytes of the basal layer can divide asymmetrically to generate cells in the more superficial epidermal layers [26]. When the basal layer was damaged, more epidermal cells were shed than produced, which is consistent with the thinning of skin thickness we observed 2 weeks after radiotherapy. This view has also appeared in the research of Wong [13]. It is worth noting that transient mild erythema may develop within hours of radiotherapy, possibly due to telangiectasia shortly after the patient’s exposure to radiation [28]. These two types of erythema may bias the assessment of the extent of radiation dermatitis.

When the radiation dose reached 30 Gy, some patients began to develop wet desquamation, which increases in frequency over the next few weeks. This is the result of the destruction and shedding of the dermis layer, accompanied by the exudation of serous fluid [26]. Previous research has suggested that edema may be responsible for the apparent increase in breast skin thickness after radiotherapy [14]. Macrophages and dermal dendritic cells within the dermis play important roles in wound healing [26]. Some scholars believed wet desquamation may be associated with long-term effects and reduced quality of life if not managed well [29].

We concluded that the increase in skin thickness at 5 weeks of radiotherapy was significantly greater in patients with severe skin reactions than milder skin reactions. In other words, we can predict the severity of acute radiation dermatitis by measuring the changes of breast skin thickness at 5 weeks of radiotherapy. Similar to our conclusions, Christopher [25] concluded that skin thickness measured by ultrasound at Week 5 was of greater significance in predicting parenchymal edema 12 months after radiation.

There was no significant change in skin elastic modulus during radiotherapy, and there was no significant difference between the two groups. This is not in line with our expectation that elastic fiber breakage caused by skin edema causes an increase in elastic modulus. Schack *et al.* [14] found that when edema is present, the dermis is markedly thickened and less echogenic under high-frequency ultrasound due to the diffusion of extracellular fluid from the subcutaneous tissue into the dermis. Adriaenssens *et al.* [16] used ultrasound elastography to compare post-operation to mean baseline measurements and reported increased subcutaneous interstitial fluid accumulation and tissue elasticity in the surgical breast after radiotherapy. Similar results were also found by Huang *et al.* [30]. In our study, the elastic modulus of the surgical side skin after radiotherapy was higher than the average value of the opposite side (17.86 ± 5.14 kpa), but there was no significant difference between the elastic modulus of the surgical side before and after radiotherapy. It may be due to the increased stiffness of the skin layer due to the hyperplasia of skin scar tissue after surgery, exceeding the effect of skin edema. Huang *et al.* [23, 31] found that the elastic modulus of keloids (124.60 kpa) was much larger than that of normal skin (17.7 kpa). In addition, we are not sure whether the radiation itself will directly damage the elastic fibers and change the skin elasticity. In the next study, the elasticity of the breast skin layer on the non-operative side of the ipsilateral breast can be included in further analysis.

Although elastography did not show a significant role in assessing skin toxicity, our study found a significant difference in skin elastic modulus in the nonsurgical quadrant before radiotherapy between the two groups. Breast skin elasticity varies with the ratio of collagen elastin to fibrous tissue [32], which may affect radiosensitivity. Further studies in more patients are needed to confirm these results.

Individual radiosensitivity is a complex trait with multifactorial and polygenic inheritance. Several patient factors may influence skin toxicity, such as breast size and BMI. Larger breasts were one of the first patient characteristics identified as a risk factor for acute skin toxicity [23, 31]. Furthermore, BMI has also been shown to be associated with the development of individual radiosensitivity. Patients with larger breasts or with a larger BMI may be more prone to wet scaling due to excessive friction [27]. Another possible explanation is that larger breast volumes have greater dose inhomogeneity compared with smaller breast volumes, which may lead to localized hot spots and subsequently increased skin toxicity.

Age is generally considered a risk factor for the development of radiation-induced skin toxicity [23, 33, 34]. However, there is conflict about its impact on the prevalence and severity of this trait. In our study, age did not affect patients’ odds of developing severe ARD. This is consistent with the findings of Liang [35] and Kraus-Tiefenbacher [36]. Garnier [23] believes that age <65 years is associated with more severe skin toxicity. In contrast, Córdoba [33] found that women over the age of 59 had a higher tendency to develop severe radiotoxicity. Other patient factors, such as the level of friction caused by normal arm movement, the texture and type of clothing worn and the accumulation of sweat, can all contribute to the skin reaction [26].

Research limitations are unavoidable. For example, the results of this study may be limited due to the small sample size and limited follow-up time (including missing data 1 week after radiotherapy). In addition, when the skin is damaged and locally inflamed, the boundary between the skin layer and the subcutaneous fat layer is not clearly displayed in the ultrasound images we used. This may cause measurement errors. Furthermore, our study only investigated the skin in a single quadrant of the breast on the radiotherapy side and did not include the effects of axillary lymph node dissection and radiation dosimetry on skin toxicity.

## CONCLUSION

Our study demonstrates that ultrasound can be used to document quantitative changes in the skin of patients with breast cancer following BCS with radiotherapy, with statistically significant differences in skin thickness between mild and severe radiation dermatitis. The study also suggested that skin elasticity may be related to individual radiosensitivity. Ultrasound can be a useful tool for non-invasive and objective assessment of skin changes during radiotherapy.

### KEY POINTS

- Ultrasonography helps clinicians to assess acute radiation dermatitis.
- Averaging of multiple point measurements provides greater diagnostic confidence.
- Treatment is given with greater confidence and patient management is more appropriate.

## CONFLICT OF INTEREST STATEMENT

The authors declare that they have no known competing financial interests or personal relationships that could have appeared to influence the work reported in this paper.
